# Ability of the So-Called Obligate Hydrocarbonoclastic Bacteria to Utilize Nonhydrocarbon Substrates Thus Enhancing Their Activities Despite their Misleading Name

**DOI:** 10.1186/s12866-019-1406-x

**Published:** 2019-02-18

**Authors:** Samir S. Radwan, Majida M. Khanafer, Husain A. Al-Awadhi

**Affiliations:** 1 Present address, Von Einem Str. 25, 48159, Münster, Germany; 2 Microbiology program, Department of Biological Sciences, Faculty of Science, Kuwait University, P.O. Box 5969, 13060 Safat, Kuwait

**Keywords:** Bioremediation, Obligate Hydrocarbonoclastic Bacteria, OHCB, *Alcanivorax*, *Marinobacter*, *Planomicrobium*

## Abstract

**Background:**

The group of the so-called obligate hydrocarbonoclastic bacteria (OHCB) are marine microorganisms affiliated with the genera *Alcanivorax*, *Cycloclasticus*, *Oleiphilus* and *Thalassolituus*. This small group plays a major role in oil-bioremediation in marine ecosystems. *Marinobacter* and *Planomicrobium* are considered related to this group. The OHCB are claimed to be obligate to hydrocarbon nutrition. This study argues against this claim.

**Results:**

Four *Alcanivorax* species, three *Marinobacter* species and *Planomicrobium okeanokoites* from the Arabian/Persian Gulf proved to be not obligate to hydrocarbon nutrition. Although the eight strains grew on crude oil, *n*-octadecane and phenanthrene as sole carbon substrates, their growth was weaker than on certain nonhydrocarbon, organic compounds viz. peptone, glutamic acid, pyruvic acid, sucrose, mannose and others. Glucose and lactose failed to support the growth of seven of the eight tested strains. Mannose was utilized by five and sucrose by six strains. The well-known intermediate metabolite; pyruvic acid was utilized by all the eight strains, and lactic acid by five strains. In batch cultures, all the tested species consumed higher proportions of peptone than of *n*-alkanes and phenanthrene. When peptone and crude oil were provided together into the medium, the OHCB started to consume peptone first, and the enriched bacterial populations consumed oil effectively. Added nonhydrocarbon substrates biostimulated oil-consumption by all OHCB species.

**Conclusion:**

The tested OHCB species are not obligate hydrocarbon-utilizers. This provides them with the merit of survival, should their marine ecosystems become oil- or hydrocarbon-free. The fact that conventional, organic substrates biostimulated hydrocarbon-consumption by the tested bacterial species confirms the facultative nature of those organisms and is interesting from the practical point of view.

**Electronic supplementary material:**

The online version of this article (10.1186/s12866-019-1406-x) contains supplementary material, which is available to authorized users.

## Background

The first reports on the so-called “obligate hydrocarbonoclastic bacteria (OHCB)” were published about two decades back [1, 2]. In definition, these are a few marine bacteria affiliated mainly to the *Proteobacteria* subclass, which are capable of growth on only two of 95 substrates of the so-called BIOLOG® system [3]. Reportedly, those two substrates are the long-chain alkyl-moiety-containing Tween 40 and Tween 80. On the other hand, nonobligate hydrocarbonoclastic bacteria utilize, in addition, many of the nonhydrocabon substrates of the BIOLOG® system. Based on that, the small group of “OHCB” was described to comprise “most highly specialized obligate hydrocarbon utilizers”, which “play a significant and global role in the natural cleansing of oil-polluted marine systems [3]”.

Systematically, the “OHCB” are affiliated to the few taxa of *Alcanivorax* spp. [2, 4–6] *Cycloclasticus oligotrophus* [7], *Oleiphilus messinensis* [8], *Oleispira antarctica* [9] and *Thallassolituus oleivorans* [10]. In addition, the nutritionally more versatile *Marinobacter* spp. are considered related to the “OHCB” [3]. The taxonomy, biography and genomic basis of ecophysiology of this group have been reviewed about one decade back [3].

Earlier investigators observed that marine systems responded to oil spills by enriching the hydrocarbonoclastic taxa named above, which are otherwise minor bacterial constituents of the pristine (oil-free) marine systems. Reportedly, the alkane-degrading *Alcanivorax* spp. were frequently the first to increase in response to oil spill, whereas *Cycloclasticus* spp. capable of degrading more complex hydrocarbons usually increased later [1, 2, 4, 11–15]. Such observations, which had been also confirmed and consolidated in microcosm-experiments [16–18] support the conclusion that the group of the so-called “OHCB” contributes effectively to the natural removal of oil spilled in the marine ecosystems. On the other hand the claimed obligate hydrocarbon nutrition of this group, could apparently represent a serious limitation to their ecological distribution in nature. Exacting microorganisms may be exposed to extinction, should their strict nutrient requirements (in this case hydrocarbons) fail in the environment. Nutritionally versatile taxa are obviously much more favored in this context.

During 25 year-research on hydrocarbonoclastic microorganisms in the permanently oil-polluted Arabian/Persian Gulf, we frequently isolated from this ecosystem taxa affiliated to the so called “OHCB” group [19–22]. We also repeatedly confirmed their role in effective removal of hydrocarbons spilled in that water body. However, the observed rich and quick growth of such bacteria on conventional, nonhydrocarbon substrates (e.g. on nutrient agar) during their isolation and subculture awaked our doubt in the validity of the term “obligate hydrocarbonoclastic”. Therefore, the major objective of this paper was to shed more light on this subject. In this contribution, we offer experimental evidence against the claimed strictly obligate hydrocarbon nutrition of *Alcanivorax* and *Marinobacter* species indigenous to the Arabian/Persian Gulf water body. We also show that such “OHCB” grow significantly better on certain nonhydrocarbon compounds than on hydrocarbon substrates as sole sources of carbon and energy. Should both types of substrates be available in the medium, these isolates start with the consumption of the nonhydrocarbon substrate first, thus amplifying their population, which is reflected in enhanced hydrocarbon utilization subsequently. As already mentioned, the strict obligate hydrocarbon requirement would deprive the concerned bacteria of an important ecophysiological merit, namely of their survival in hydrocarbon-free (pristine) niches.

## Results

Eight hydrocarbonoclastic bacterial species from the Arabian/Persian Gulf coastal water belonging or related to the “OHCB” were found to utilize also nonhydrocarbon (conventional) substrates, some of which even more effectively than hydrocarbon substrates.

### Strains of “OHCB” from the Arabian/Persian gulf

**Fig. 1:**
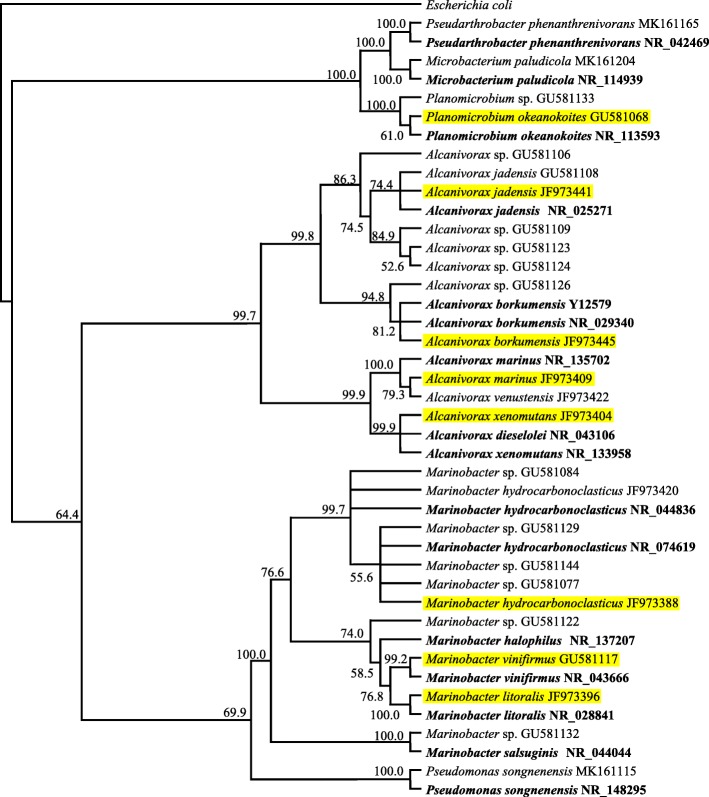
A phylogenetic tree illustrating the relationships among all the “OHCB” isolated from the Arabian Gulf in our laboratory. The tree is constructed by neighbor-joining method. Numbers at nodes indicate bootstrap values of 2000 resembling; 0.1 denotes the genetic distance. Strains in bold are type strains from the GenBank database; highlighted strains are the “OHCB” used in this study; the remaining are hydrocarbonoclastic strains isolated in our laboratory. Three nonobligate hydrocarbonoclastic species, *Microbacterium paludicola*, *Pseudomonas songnenensis* and *Arthrobacter phenanthrenivorans* were included for comparison

Bacterial strains of the “OHCB” used in this contribution had been isolated in our laboratory from sampling sites along the Arabian Gulf as shown in Table 1 (see also the Kuwait map in the Additional file 1: Figure S1.). Four strains were affiliated to *Alcanivorax*, three to *Marinobacter* and one to *Planomicrobium okeanokoites*. Their 16S rRNA-gene sequences showed 99 to 100% similarities to the sequences of type strains in the GenBank database. The phylogenetic relationships among those “OHCB” are illustrated in Fig. 1. For comparison, three nonobligate hydrocarbonoclastic species, *Microbacterium paludicola*, *Pseudomonas songnenensis* and *Arthrobacter phenanthrenivorans* were included in the tree.

**Table 1:** Sources of “OHCB” from the Arabian Gulf and information about their 16S rDNA-sequencing

Hydrocarbonoclastic strain (accession no.)	Source	Nearest GenBank Match [bases compared (bp), % similarity] (class, accession no.)	References
*Alcanivorax* sp. (JF973445)	Az Zour (coastal water)	*Alcanivorax borkumensis* [524, 100] (γ-P, NR_029340)	21
*Alcanivorax* sp. (JF973404)	Sharq (coastal water)	*Alcanivorax xenomutans* [513, 100] (γ-P, NR_133958)	21
*Alcanivorax* sp. (JF973441)	Al-Khiran (coastal water)	*Alcanivorax jadensis* [513, 100] (γ-P, NR_025271)	21
*Alcanivorax* sp. (JF973409)	Sharq (coastal water)	*Alcanivorax marinus* [506, 99] (γ-P, NR_135702)	21
*Marinobacter* sp. (JF973388)	Doha (coastal water)	*Marinobacter hydrocarbonoclasticus* [513, 100] (γ-P, NR_074619)	21
*Marinobacter* sp. (JF973396)	Doha (coastal water)	*Marinobacter litoralis* [509, 99] (γ-P, NR_028841)	21
*Marinobacter* sp. (GU581117)	Doha (Epilithic biofilm on gravel)	*Marinobacter vinifirmus* [508, 99] (γ-P, NR_043666)	20
*Planomicrobium* sp. (GU581068)	Anjefa (Epilithic biofilm on gravel)	*Planomicrobium okeanokoites* [496, 99] (Bac, NR_113593)	20

γ-P,γ-Proteobacteria; Bac, Bacilli

### Bacterial growth on hydrocarbon and nonhydrocarbon substrates

**Fig. 2:**
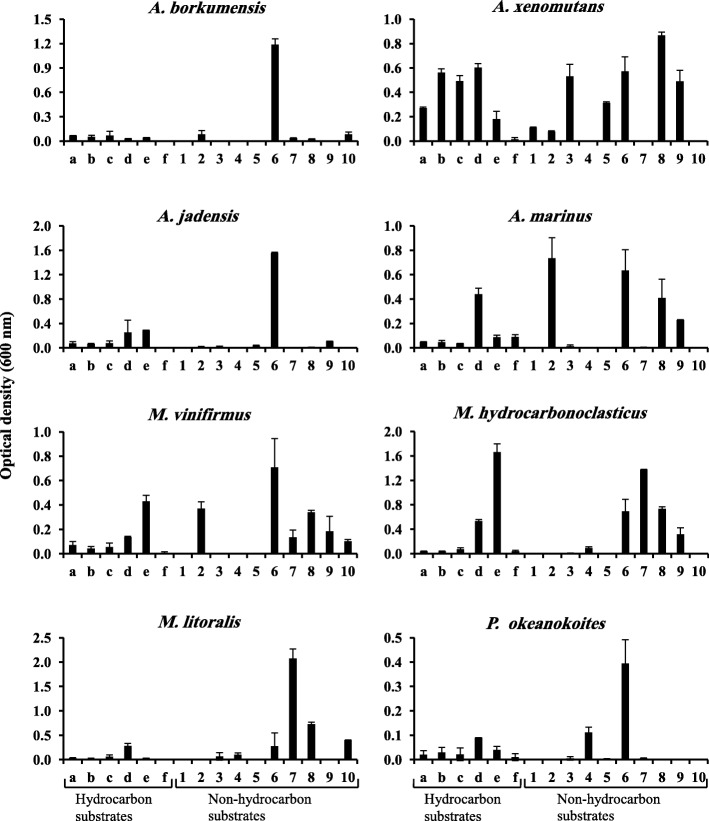
Growth of eight “OHCB” and related strains from the Arabian Gulf on various substrates as sole sources of carbon and energy. The substrate concentration was 500 mg l^− 1^. Incubation was for 12 d at 30 °C. Each reading is the mean of 3 replicates. In several cases the growth on nonhydrocarbon substrates was even better than on typical hydrocarbon substrates and Tween 80. Error bars represent the standard error based on the three measurements Substrates: **a**, crude oil; **b**, *n*-octadecane; **c**, phenanthrene; **d**, Tween 80; **e**, stearyl alcohol; **f**, oleic acid; 1,glucose; 2, mannose; 3, sucrose; 4, starch; 5, glycerol; 6, peptone; 7, glutamic acid; 8, pyruvic acid; 9, lactic acid; 10, citric acid

The term “obligate hydrocarbonoclastic” implies that the so designated species utilize hydrocarbon substrates but fail to utilize nonhydrocarbon substrates. The histograms in Fig. 2 show that the eight tested bacterial species grew successfully on the typical hydrocarbons; crude oil, *n*-octadecane and phenanthrene as sole sources of carbon and energy. Significantly better growth (*p* < 0.05) was however recorded for *A*. *xenomutans* on the aliphatic and aromatic hydrocarbons (*n*-octadecane and phenanthrene, respectively) than on the other hydrocarbon substrates. On Tween 80; one of the constituent substrates of the BIOLOG® system [3], the growth of all the *Alcanivorax* and *Marinobacter* species was even significantly better than on the typical hydrocarbon substrates (*p* < 0.05) probably due to the ready solubility of Tween 80 in water. Based on that, the affiliation and relationship of those species to the “OHCB”, as defined by earlier investigators is justified, although of course their ability to utilize nonhydrocarbon substrates contradicts the term “obligate” [3]. Only *P*. *okeanokoites* failed to grow on Tween 80 which may suggest excluding it from the “OHCB” group although it may still remain related to it. Interestingly, the phylogenetic tree (Fig. 1) shows that this species had the remotest genetic relationship to all the remaining species. Stearyl alcohol significantly (*p* < 0.05) supported the growth of the eight tested strains particularly of *M. hydrocarbonoclasticus* and, to a less extent of *M*. *vinifirmus*. Oleic acid supported albeit rather weak growth of three species only viz. *A*. *xenomutans*, *A. marinus* and *M. hydrocarbonoclasticus* but failed to support growth of any other species. Within this context, the initial attack of hydrocarbonoclastic microorganisms on alkane substrates involves their hydroxylation to the corresponding alcohols and fatty acids which are subsequently catabolized by β-oxidation leading to the key intermediate metabolite, acetyl CoA [23–25]. Through the tricarboxylic acid cycle, the latter key metabolite is used for the production of cell materials and ATP. Based on these facts, it is surprising that *A*. *borkumensis*, *A*. *jadensis*, *M*. *vinifirmus*, *M. litoralis* and *P*. *okeanokoites* grew successfully on the alkane *n*-octadecane but failed to grow on its oxidation product, oleic acid. Possibly those strains do not possess the mechanism(s) with which they can take up fatty acids.

Compared with the individual hydrocarbon substrates, the tested carbohydrate substrates were mostly less effective in serving as sole sources of carbon and energy for the eight tested “OHCB”. Thus, glucose failed to support the growth of seven of the tested species and only *A*. *xenomutans* showed rather weak growth on this sugar. Mannose on the other hand, surprisingly supported weak growth of *A*. *borkumensis* (the paradigm of “OHCB”, [3], *A*. *xenomutans* and *A*. *jadensis* but also rather good growth of *A. marinus* and *M*. *vinifirmus* (*p* < 0.05). The disaccharide, sucrose supported weak growth of five of the tested species viz. *A*. *jadensis*, *A. marinus*, *M. hydrocarbonoclasticus*, *M. litoralis* and *P*. *okeanokoites* and rather good growth of *A*. *xenomutans* (*p* < 0.05). On the other hand, lactose failed to support the growth of any of the eight tested species (not shown in the histogram). All the *Alcanivorax* species failed to grow on the polysaccharide, starch as sole source of carbon and energy, but the two *Marinobacter* species, *M. hydrocarbonoclasticus* and *M. litoralis* as well as *P*. *okeanokoites* could grow on this polysaccharide as a carbon substrate, albeit rather weakly. Glycerol supported fair growth of *A*. *xenomutans* and *A*. *jadensis* (*p* < 0.05). Although monosaccharides, disaccharides and polysaccharides as well as glycerol did not serve as excellent carbon sources, the mere growth of the tested “OHCB” on many of those nonhydrocarbon substrates sheds doubt on considering those organisms “obligate” hydrocarbonoclastic.

The eight tested species grew fairly well to excellently (especially *A*. *borkumensis* and *A*. *jadensis*) on the nonhydrocarbon substrate, peptone. The optical density values with this substrate were in most cases even higher than the corresponding values with crude oil, *n*-octadecane, phenanthrene or Tween 80 as sole carbon and energy sources (*p* < 0.05). Two of the tested strains; *A*. *borkumensis* (the paradigm of “OHCB”) and *P*. *okeanokoites*, grew weakly, and three; *M*. *vinifirmus*, *M. hydrocarbonoclasticus* and *M. litoralis* grew rather well (*p* < 0.05) on glutamic acid as a sole source of carbon and energy. None of the eight strains could grow on the amino acid; tryptophan as a carbon substrate (not shown in the histogram).

Out of the three tested intermediate metabolites, pyruvic acid was the only substrate utilized by the eight tested strains. Within this context, an earlier study in our laboratory revealed that pyruvate-utilizing bacteria were contributors to the food web in the Arabian/Persian Gulf [26]. Weak growth of *A*. *borkumensis* and *A*. *jadensis*, but significantly better growth of *A*. *xenomutans*, *A. marinus*, *M*. *vinifirmus*, *M. hydrocarbonoclasticus*, *M. litoralis* and *P*. *okeanokoites* occurred on this substrate as a sole source of carbon and energy (*p* < 0.05). Lactic acid supported fair growth of five of the tested strains, namely *A*. *xenomutans*, *A*. *jadensis*, *A. marinus*, *M*. *vinifirmus* and *M. hydrocarbonoclasticus* (*p* < 0.05). It is well known that lactic acid become dehydrogenated biologically leading to pyruvic acid. Citric acid also supported growth of *A*. *borkumensis*, *M*. *vinifirmus* and *M. litoralis*. Based on the substrate-utilization patterns in Fig. 2, *A*. *borkumensis* and *A*. *jadensis* are almost identical, but quite different from *A. marinus* and *A*. *xenomutans*. Surprisingly, the latter *Alcanivorax* species is more versatile in its nutrition than all the other 7 species including those reported as “related to the OHCB”.

The growth qualities of three *Alcanivorax* species and two *Marinobacter* species on the solid mineral medium with crude oil and some nonhydrocarbon substrates as sole sources of carbon and energy are illustrated in the Additional file 1: Figure S2. The results quite clearly indicate that in many cases, growth on the conventional substrates was even richer than on crude oil.

**Fig. 3:**
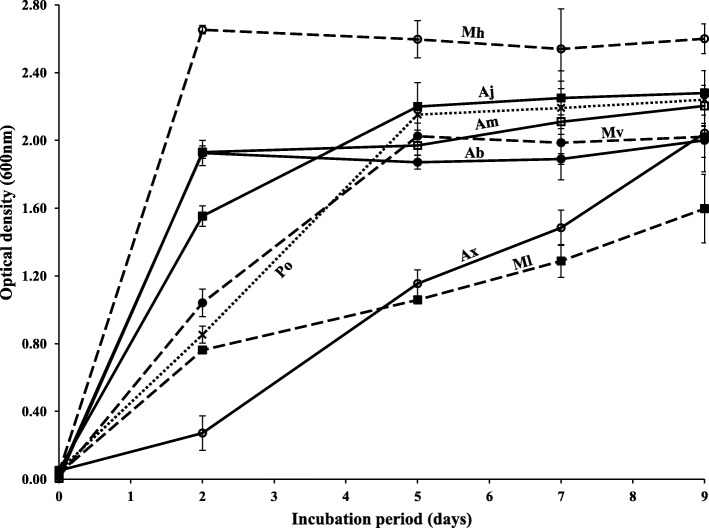
Growth curves of “OHCB” from the Arabian Gulf in a mineral medium with 500 mg l^− 1^, peptone as a sole source of carbon and energy. Each reading was the mean of 3 replicates. Error bars represent the standard error based on the three measurements. Ab, *A*. *borkumensis*; Ax, *A*. *xenomutans*; Aj, *A*. *jadensis*; Am, *A. marinus*; Mv, *M*. *vinifirmus*; Mh, *M*. *hydrocarbonoclaticus*; Ml, *M. litoralis*; Po, *P*. *okeanokoites*

In view of the fact that peptone proved to be a utilizable substrate by the eight tested “OHCB” (Fig. 2), growth curves were constructed for those bacteria using a mineral medium with peptone as a sole source of carbon and energy. The results in Fig. 3 show that the eight tested bacterial species grew well in that medium. Most of the growth occurred during the first few days prior to the onset of the stationary growth phases. The rich growth of those “OHCB” is also well demonstrated by the turbidities of the used liquid peptone media at the end of the incubation period (Additional file 1: Figure S3.).

### Preferred utilization of nonhydrocarbon substrates by “OHCB”

**Fig. 4:**
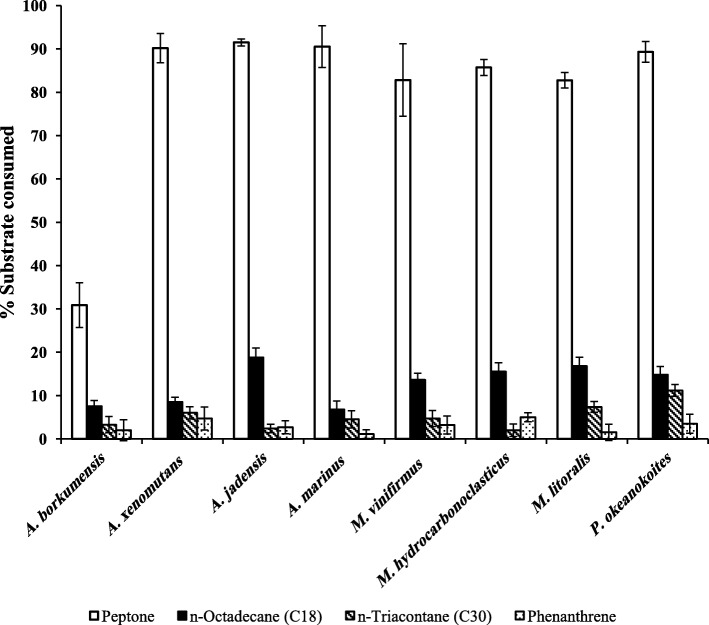
Consumption of higher proportions of peptone than of hydrocarbons by “OHCB” from the Arabian Gulf. The concentration of the tested substrates was 500 mg l^− 1^. Error bars represent the standard error based on three measurements

Figure 4 shows that the eight tested bacterial species consumed more proportions of peptone than of any of the three pure hydrocarbons tested (Additional file 1: Figure S4. shows the standard curve used for peptone determination). The lowest proportion of peptone, (31%) was consumed by the “OHCB paradigm”; *A*. *borkumensis*. To recall, the mere consumption of peptone by this organism reveals its “facultative” hydrocarbon mode of nutrition. The remaining seven species consumed between 83 and 92% of the available peptone. Among the tested hydrocarbons, the best utilized one was C_18_ whose consumption proportions ranged between 8 and 17%. The *n*-alkane, C_30_, with consumption proportions between 2 and 11% was less readily consumed and phenanthrene with consumption proportions of only 2 to 5% was the least readily utilized substrate by the eight tested bacterial species. This result provides an experimental evidence that “OHCB” from the Arabian/Persian Gulf (probably from elsewhere too) may prefer peptone over hydrocarbons as a carbon source.

**Fig. 5:**
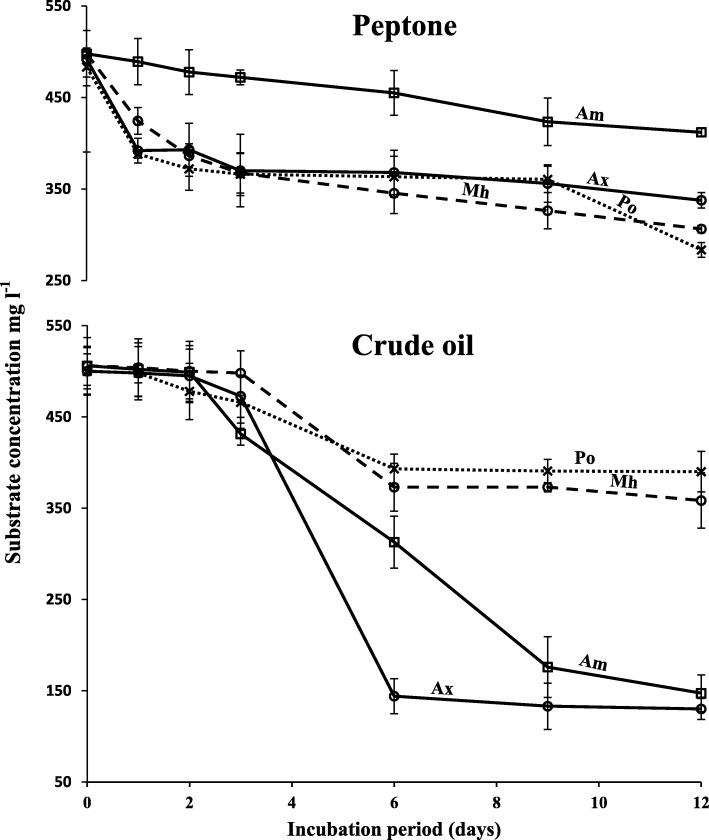
Utilization of a mixture of peptone and crude oil by “OHCB” from the Arabian Gulf. The substrate concentrations were 500 mg l^− 1^. Error bars represent the standard error based on three measurements. Ax, *A*. *xenomutans*; Am, *A. marinus*; Mh, *M*. *hydrocarbonoclaticus*; Po, *P*. *okeanokoites*

In another experiment, the nonhydrocarbon substrate; peptone and the hydrocarbon substrate; crude oil (a mixture of numerous hydrocarbons), 500 mg l^− 1^, each were provided together in the mineral medium. Their consumption was measured through a 12 day incubation period at 30 °C. Additional file 1: Figure S5. shows the typical GLC profiles illustrating the crude-oil consumption. The results in Fig. 5 indicate that peptone consumption by all the tested species occurred effectively in the first few days, just before any crude oil started to be consumed. However, crude oil consumption started later (after day 4) and was rather vigorous at day 6, which suggests that the bacteria prefer crude oil after they acquire nutrients from peptone.

### Effects of nonhydrocarbon substrates on bacterial growth and oil-consumption

**Fig. 6:**
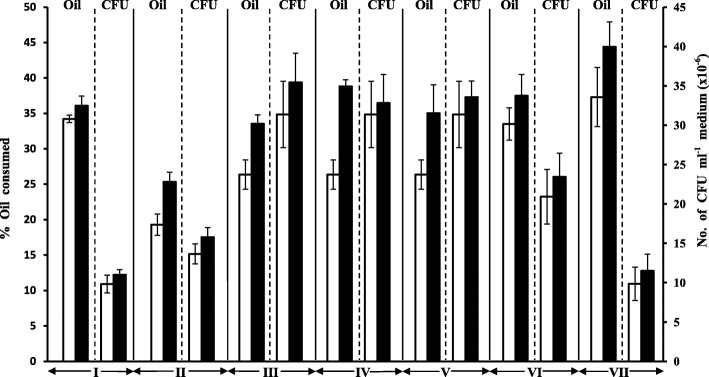
Effects of nonhydrocarbon substrates on oil-consumption values and numbers of bacterial cells. Open columns, flasks containing oil only (controls); closed columns, flasks containing oil and one nonhydrocarbon substrate. Error bars represent the standard error based on three measurements. I, *A*. *borkumensis*/peptone; II, *A*. *jadensis*/peptone; III, *A*. *xenomutans*/pyruvic acid; IV, *A*. *xenomutans*/lactic acid; V, *A*. *xenomutans*/sucrose; VI, *M*. *hydrocarbonoclaticus*/glutamic acid; VII, *M*. *vinifirmus*/mannose

Figure 6 shows that the four *Alcanivorax* spp. and the two *Marinobacter* spp. studied grew better and consumed more oil in cultures supplied with nonhydrocarbon compounds than in control cultures without those substrates (*p* < 0.05 in all cases). This response was most pronounced for *A*. *xenomutans* supplied with lactic acid and sucrose and *M*. *vinifirmus* supplied with mannose where the oil-consumption increased from 26.4 to 38.8%, from 26.4 to 35.0% and from 37.3 to 46.4%, respectively. It may appear contradictory that lactic acid which yielded relatively poor growth caused comparatively effective enhancement of oil-consumption. Probably the involved species have such a high oil-utilization potential that fewer cells are effective in oil-removal than more cells of species with less hydrocarboboclastic potential. Less, albeit still considerable responses were measured for *A*. *jadensis* supplied with peptone, *A*. *xenomutans* supplied with pyruvic acid and *M. hydrocarbonoclasticus* supplied with glutamic acid, where the oil-consumption increased from 19.3 to 25.3%, from 26.4 to 33.5% and from 33.5 to 37.4%, respectively. The typical GLC-profiles in the Additional file 1: Figure S6 illustrate some of those interesting results described above.

## Discussion

The *Alcanivorax* species included *A*. *borkumensis*, which has been designated by earlier investigators as the “paradigm” of “OHCB” [3]. The remaining three species comprised *A*. *xenomutans* [6], *A*. *jadensis* [14, 27]; and *A. marinus* [5], which were also reported in the literature as typical “OHCB”. The *Marinobacter* species comprised *M. hydrocarbonoclasticus*, which is related to the “OHCB”and is synonymous with *M*. *aquaeolei* (*Pseudomonas nautical*) [3]. The other two *Marinobacter* species; *M. litoralis* and *M*. *vinifirmus*, although also hydrocarbonoclastic [21], were not recorded in the available literature as “OHCB”. Also the hydrocarbonoclastic strain *Planomicrobium okeanokoites* [28] has not been reported so far as “OHCB”, although another species of the same genus, *P*. *alkanoclasticum* has been considered related to this group [3]. In other words, at least the four *Alcanivorax* spp. are commonly accepted as typical “OHCB”. The remaining four species are considered related to this group. As mentioned above, typical “OHCB” were considered so when they grew on Tween 40 and Tween 80 only out of the 95 substrates of the BIOLOG® system.

The findings of this study imply that the “OHCB” from the Arabian Gulf (probably from elsewhere too) can utilize beside hydrocarbons also nonhydrocarbon substrates. This result sheds doubt on the claimed obligate hydrocarbon mode of nutrition of the “OHCB”, but it is meanwhile of ecophysiological significance, even though the growth on some conventional substrates was weak. Even weak growth is enough for the concerned microorganism to survive, should hydrocarbon substrates fail in the open environment.

As already mentioned, it is commonly believed that “OHCB”, in contrast to other bacteria, are specialized utilizers of a very narrow group of organic substrates [3]. It has also been reported that *Marinobacter* spp. are nutritionally more versatile than *Alcanivorax* spp. However, the results in Fig. 2 do not support these claims. Out of the 19 tested substrates, 11 were successfully utilized by each of *A*. *jadensis*, *A. marinus*, *M*. *vinifirmus* and *M. litoralis*, 12 by *M. hydrocarbonoclasticus* and 14 by *A*. *xenomutans*. The lowest substrate numbers were utilized by *A*. *borkumensis* (10 substrates) and *P*. *okeanokoites* (8 substrates). In other words the eight tested organisms actually utilized relatively high proportions of the 19 tested substrates, namely, 42, 53, 60, 63 and 73% by *P*. *okeanokoites*, *A*. *borkumensis* (the paradigm of “OHCB”), *A*. *jadensis*, *M. hydrocarbonoclasticus* and *A*. *xenomutans*, respectively. These findings also oppose the claim that those bacterial species could “utilize only a very narrow group of organic substrates”.

The preferred utilization of peptone over hydrocarbons by the isolates may be attributed to three probable factors. The first is the slower availability and utilizability of the water insoluble crude oil than the water soluble peptone. The second is the pre-enrichment of the bacterial population in response to the preferred utilization of peptone, which was subsequently reflected in more vigorous crude oil consumption. Such a synergistic behavior is interesting and should be considered during designing bioremediation technologies for oil contaminated environments. The third factor is that peptone in contrast to hydrocarbon substrates serves not only as a source of carbon and energy, but also as a source of nitrogen. Nitrogen fertilizers are long known to enhance microbial hydrocarbon consumption [20, 23–25].

The mere enhancement of growth and oil-consumption by the studied strains when provided with nonhydrocarbon substrates is another experimental evidence against the claimed obligate hydrocarbon nutrition of those bacterial species. From the practical view point, the enhanced hydrocarbon-consumption is obviously interesting when biotechnologies for remediating oily marine ecosystems (probably other ecosystems too) are suggested.

## Conclusions

In conclusion, “OHCB” from the Arabian Gulf are not strictly obligate to hydrocarbon substrates as claimed in the literature. Differences in environmental physiochemical parameters could be argued to be responsible for such contradiction. However, inhabiting a hydrocarbon-contaminated water body since geological ages, the Arabian/Persian Gulf strains should expectedly be among the most obligate strains to hydrocarbon nutrition worldwide. Within this context, the obligate hydrocarbonoclastic activity is not a merit for the concerned microorganism. On the contrary, should the environment become free of hydrocarbons for one reason or another, such exacting “OHCB” would be exposed to extinction. Survival of the so-called “OHCB” with even weak growth rates in the presence of nonhydrocarbon (conventional) substrates, as the results of this study demonstrate, saves them from potential extinction. Our finding that nonhydrocarbon substrates biostimulate oil-removal by hydrocarbonoclastic bacteria is obviously interesting from the practical point of view.

## Methods

### The tested “obligate hydrocarbonoclastic bacteria”

The eight “obligate hydrocarbonoclastic” bacterial species used in this study (listed in Table 1) had been isolated previously in our laboratory from the Arabian/Persian Gulf coastal waters [20, 21] using a selective medium. This was a mineral medium with crude oil vapor as a sole source of carbon and energy [29]. It consisted of (g l^− 1^): 30.0 NaCl, 5.0 NaNO_3_, 0.56 KH_2_PO_4_, 0.86 Na_2_HPO_4_, 0.17 K_2_SO_4_, 0.37 MgSO_4_.7H_2_O, 0.007 CaCl_2_.H_2_O; and 25 ml l^− 1^ of a trace element solution consisting of (g l^− 1^): 2.32 CuSO_4_.5H_2_O, 0.39 Na_2_MoO_4_. 2H_2_O, 0.66 KI, 1.0 EDTA, 0.4 FeSO_4_.7H_2_O, 0.004 NiCl_2_.6H_2_O. The pH was adjusted to 7 using N/10 NaOH or N/10 HCl. For solidification, 2% agar was added. The isolated bacterial strains had been characterized by comparing the sequences of their 16S rRNA-genes with those of type strains in the Genbank database. For this, total genomic DNA was first extracted from the cells using the PrepMan Ultra Sample Preparation Reagent (Applied Biosystems, USA) following the producer instructions. The 16S rRNA-genes were amplified by polymerase chain reaction (PCR) using the universal bacterial primer combination, GM5F and 907R [30]. The PCR products were purified using the QIAquick PCR Purification Kit (Applied Biosystems, USA). The pure DNA samples were processed in a 3130*xl* genetic analyzer using Sequencing analysis version 5.2 software. Sequences were subjected to basic local alignment search tool analysis with the National Center for Biotechnology Information (NCBI, USA) GenBank database [31]. A phylogenetic tree illustrating the relationships among the eight “OHCB” from the Arabian Gulf was constructed by a neighbor joining analysis using PAUP* v.4 [32]. Bootstrap proportions were used on 2000 replicates. The four *Alcanivorax* spp. used were recognized by earlier investigators [3, 5, 6, 8] as “OHCB”, whereas the three *Marinobacter* spp. in addition to *Planomicrobium* sp. were considered related to this group [3].

### Bacterial growth on hydrocarbon and nonhydrocarbon substrates

Growth qualities of the studied bacterial species on solid mineral medium provided with 500 mg l^− 1^ of the tested substrates were examined. One loopful portions of common inocula of individual bacteria were streaked on the solid mineral medium surface. Triplicates were prepared and the cultures were incubated at 30 °C for 12 d and examined for growth. The substrates studied comprised hydrocarbons and related products as well as nonhydrocarbon compounds. The former group included light Kuwaiti crude oil (Kuwait Oil Company), *n*-octadecane (C_18_), phenanthrene, Tween 80, stearyl alcohol and oleic acid (purchased from Sigma-Aldrich, USA). The second group comprised carbohydrates and related compounds viz. glucose, mannose, lactose, sucrose, starch and glycerol (purchased from Himedia, India), polypeptides and amino acids viz. peptone (purchased from Oxoid, UK), glutamic acid and tryptophan (purchased from Sigma-Aldrich, USA) and intermediate metabolites viz. pyruvic acid, lactic acid and citric acid (purchased from Sigma-Aldrich, USA).

Bacterial growth in 20 ml aliquots of the liquid mineral medium containing hydrocarbon and/or nonhydrocarbon substrates was quantitatively measured in terms of optical densities (Absorbance) at 600 nm using a spectrophotometer (Thermo Spectronic Genesys 5, USA). For this, the liquid medium aliquots containing the substrates (500 mg l^− 1^) were inoculated with 0.2 ml aliquots of common inocula of individual bacteria and incubated on an electrical shaker, 120 rpm, at 30 °C for 12 d. Triplicates were prepared throughout.

To construct growth curves, it was proceeded as described above, and the optical densities of the media were measured in different time intervals. Also here, triplicate readings in three parallel experiments were measured.

### Preferred utilization of nonhydrocarbon substrates by “OHCB”

The objective of these experiments was to find out which sort of substrates is preferred by the studied bacterial species, should hydrocarbon and nonhydrocarbon (conventional) substrates be available simultaneously. Liquid mineral medium aliquots, 100 ml, were dispensed in 250 ml conical flasks. Each flask received one hydrocarbon and one nohydrocarbon substrates at the concentration of 500 mg l^− 1^, each.

The hydrocarbon substrates studied were light crude oil, pure *n*-alkanes with C_18_ (*n*-octadecane) and C_30_ (*n*-triacontane) chains as well as pure phenanthrene. As a nonhydrocarbon substrate, peptone was selected because it was found to be utilizable by all the tested species. Triplicates were prepared throughout. The cultures were incubated on an electrical shaker, 120 rpm, at 30 °C for 12 d. The residual hydrocarbons in each flask were extracted and determined and the residual peptone in the aqueous phase was also quantitatively determined (see below).

To compare the utilization kinetics when hydrocarbon and nonhydrocarbon (conventional) substrates were simultaneously available, the mineral medium was provided with 500 mg l^− 1^ each of light crude oil and peptone. The cultures were inoculated and incubated on an electrical shaker, 120 rpm, at 30 °C for 12 d, as already described. At different time intervals, cultures in triplicates were harvested for quantitative determination of residual oil and peptone.

### Quantitative determination of crude oil

The residual crude oil was recovered from the medium by three successive extractions with 10 ml aliquots of pentane. The volume of the combined extract was completed to 35 ml with pure pentane. Aliquots of 1.0 μl were analyzed by gas liquid chromatography (GLC) using an Aglient 7890A GC (USA) equipment provided with a flame ionization detector (FID), a DB-5 capillary column (Aglient Technologies, USA) and He as a carrier gas. The oven temperature program started at 50 °C for 3 min then rising at 3 °C/min to 80 °C then rising at 8 °C/min to 256 °C then rising at 30 °C/min to 330 °C and holding at this temperature for 11 min.. The percentage of oil consumption was determined by calculating the reduction of the total peak areas in the GLC profiles based on the peak areas of crude oil recovered from time-zero, control samples.

### Quantitative determination of peptone

The classical Biuret Protein Assay [33] was adopted for the determination of residual peptone in the liquid culture media. Aliquots, 1.0 ml of each sample were pipetted into test tubes containing 4.0 ml of Biuret reagent consisting of (g l^− 1^) 9.0 NaKC_4_H_4_O_6_. 4H_2_O, 3.0 CuSO_4_.5H_2_O, 5.0 KI and 400 ml 0.2 M NaOH. The tubes were vortexed and kept at room temperature for 20 min. Triplicates were prepared throughout. The optical densities were measured at 540 nm using a spectrophotometer (Thermo Spectronic Genesys 5, USA). For the construction of the calibration curve, a series of mineral medium aliquots containing known concentrations of peptone (1.0, 1.5, 2.0, 2.5, 3.0, 3.5, 4.0, 4.5, 5.0, 7.0 and 10.0 mg l^− 1^) was prepared. The prepared medium aliquots were incubated with Biuret reagent and the optical densities at 540 nm measured. The calibration curve was used to read the concentrations of residual peptone in the studied bacterial cultures.

### Effects of nonhydrocarbon substrates on bacterial growth and oil-consumption

The objective of this experiment was to test the validity of the assumption that the easily utilizable nonhydrocarbon substrates may amplify the hydrocarbonoclastic population thus leading to enhanced oil-consumption.

Mineral medium aliquots, 50 ml, were dispensed in 250 ml conical flasks and provided with 25 mg aliquots (500 mg l^− 1^) of crude oil. Some flasks received in addition 25 mg aliquots of peptone, others of pyruvic acid, lactic acid, glutamic acid, mannose or sucrose. The peptone-flasks were separately inoculated with *A*. *borkumensis* and *A*. *jadensis*, the pyruvic acid-, lactic acid- and sucrose-flasks with *A*. *xenomutans*, the glutamic acid-flasks with *M. hydrocarbonoclasticus* and the mannose-flasks with *M*. *vinifirmus*. Control flasks containing only oil but free of any added nonhydrocarbon substrates were also inoculated. Triplicates were prepared throughout and the cultures were incubated on an electrical shaker, 120 rpm, at 30 °C for 8 d. The colony forming units (CFU) numbers were counted and the residual oil extracted and determined by GLC as described above. The mean values were calculated and the results were represented as histograms.

### Statistical analysis

Three determinations for each analysis were done and the mean values ± standard deviation values were calculated using Microsoft Excel 2007. Also, Statistical Package for Social Sciences, version 12 was used to assess the degree of significance, where the analysis of variance (ANOVA) was used to differentiate between the means of the tested parameters.

## Additional file

Additional file 1: **Figure S1.** Kuwait map showing the water sampling sites (source: d-maps.com: https://d-maps.com/carte.php?num_car=400&lang=en, labeled by Microsoft Office 2007). **Figure S2.** Comparison of growth qualities for *Alcanivorax* spp. and *Marinobacter* spp. from the Arabian Gulf on solid mineral medium containing crude oil or conventional carbon sources (500 mg l^− 1^) as sole sources of carbon and energy. A, *A*. *borkumensis*; B, *A*. *dieselolei*; C, *A. marinus*; D, *M. hydrocarbonoclasticus*; E, *M*. *vinifirmus* . **Figure S3.** Liquid cultures of individual OHCB from the Arabian Gulf in mineral medium supplied with 500 mg l^− 1^ peptone. as a sole source of carbon and energy. A, the *Alcanivorax* spp., 1, *A*. *xenomutans*; 2, *A*. *jadensis*; 3, abiotic control (not inoculated medium); 4, *A. marinus*; 5, *P*. *okeanokoites*; B, the *Marinobacter* spp., 1, *M. hydrocarbonoclasticus*; 2, *M*. *vinifirmus*; 3, *M. litoralis*; 4, abiotic control. The medium turbidity indicates the excellent peptone utilization by the studied OHCB. **Figure S4.** Standard curve constructed for determination of peptone. **Figure S5.** GLC profiles showing crude oil consumption by individual OHCB from the Arabian Gulf. **Figure S6.** Typical GLC-profiles illustrating the enhancement of growth and oil-consumption by representative “OHCB” when treated with nonhydrocarbon substrates. Smaller peaks mean less residual oil in the medium due to higher oil-consumption rates by the tested strain. (PDF 592 kb)

## Abbreviations

ANOVA: Analysis of variance
CFU: Colony forming units
FID: Flame ionization detector
GLC: Gas liquid chromatography
OHCB: Obligate hydrocarbonoclastic bacteria
PCR: polymerase chain reaction

## References

[1] Yakimov MM, Golyshin PN, Lang S, Moore ER, Abraham WR, Lünsdorf H, Timmis KN (1998). Alcanivorax borkumensis gen nov, sp nov, a new, hydrocarbon-degrading and surfactant producing marine bacterium. Int J Syst Bacteriol.

[2] Golyshin PN, Harayama S, Timmis KN, Yakimov MM, Alcanivoraceae F, Garrity G (2005). Bergey’s manual of systematic bacteriology.

[3] Yakimov MM, Timmis KN, Golyshin PN (2007). Obligate oil-degrading marine bacteria. Curr Opin Biotech.

[4] Dyksterhouse SE, Gray JP, Herwig RP, Lara JC, Staley JT (1995). Cycloclasticus pugetii gen nov, sp nov, an aromatic hydrocarbon-degrading bacterium from marine sediments. Int J Syst Bacteriol.

[5] Lai Q, Wang J, Gu L, Zheng T, Shao Z (2013). *Alcanivorax marinus* sp nov, isolated from deep-sea water. Int J Syst Evol Microbiol.

[6] Rahul K, Sasikala C, Tushar L, Debadrita R, Ramana CV (2014). *Alcanivorax xenomutans* sp nov, a hydrocarbonoclastic bacterium isolated from a shrimp cultivation pond. Int J Syst Evol Microbiol.

[7] Robertson BR, Button DK, Koch AL (1998). Determination of the biomasses of small bacteria at low concentrations in a mixture of species with forward light scatter measurements by flow cytometry. Appl Environ Microbiol.

[8] Golyshin PN, Chernikova TN, Abraham WR, Lünsdorf H, Timmis KN, Yakimov MM (2002). *Oleiphilaceae* fam nov, to include Oleiphilus messinensis gen nov, sp nov, a novel marine bacterium that obligately utilizes hydrocarbons. Int J Syst Bacteriol.

[9] Yakimov MM, Giuliano L, Gentil G, Crisafi E, Chernikova TN, Abraham WR, Lünsdorf H, Timmis KN, Golyshin PN (2003). Oleispira Antarctica gen nov, sp nov, a novel hydrocarbonoclastic marine bacterium isolated from Antarctic coastal sea water. Int J Syst Evol Microbiol.

[10] Yakimov MM, Giuliano L, Denaro R, Crisafi E, Chernikova TN, Abraham WR, Lünsdorf H, Timmis KN, Golyshin PN (2004). Thalassolituus oleivorans gen nov, sp nov, a novel marine bacterium that obligately utilizes hydrocarbons. J Syst Evol Microbiol.

[11] Harayama S, Kishira H, Kasai Y, Shutsubo K (1999). Petroleum biodegradation in marine environments. J Mol Microbiol Biotechnol.

[12] Kasai Y, Kishira H, Sasaki T, Syutsubo K, Watanabe K, Harayama S (2002). Predominant growth of *Alcanivorax* strains in oil-contaminated and nutrient-supplemented sea water. Environ Microbiol.

[13] Kasai Y, Kishira H, Harayama S (2002). Bacteria belonging to the genus *Cycloclasticus* play a primary role in the degradation of aromatic hydrocarbons released in a marine environment. Appl Environ Microbiol.

[14] Golyshin PN, Martins Dos Santos VA, Kaiser O, Ferrer M, Sabirova YS, Lünsdorf H, Chernikova TN, Golyshina OV, Yakimov M (2003). Pühler a, Timmis KN. Genome sequence completed of *Alcanivorax borkumensis*, a hydrocarbon degrading bacterium that plays a global role in oil removal from marine systems. J Biotechnol.

[15] Head IM, Jones DM, Roling WF (2006). Marine microorganisms make a meal of oil. Nat Rev Microbiol.

[16] Harayama S, Kasai Y, Hara A (2004). Microbial communities in oil-contaminated seawater. Curr Opin Biotechnol.

[17] Yakimov MM, Denaro R, Genovese M, Cappello S, D'Auria G, Chernikova TN, Timmis KN, Golyshin PN, Giluliano L (2005). Natural microbial diversity in superficial sediments of Milazzo Harbor (Sicily) and community successions during microcosm enrichment with various hydrocarbons. Environ Microbiol.

[18] Cappello S, Denaro R, Genovese M, Giuliano L, Yakimov MM (2007). Predominant growth of *Alcanivorax* during experiments on ‘oil spill bioremediation’ in mesocosms. Microbiol Res.

[19] Al-Mailem,D, Sorkhoh N, Salamah S, Eliyas M, Radwan SS. Oil-bioremediation potential of Arabian gulf mud flats rich in diazotrophic hydrocarbon-utilizing bacteria. Int Biodeter Biodegr. 2010;64:218–225.

[20] Radwan SS, Mahmoud H, Khanafer M, Al-Habib A, Al-Hasan R (2010). Identities of epilithic hydrocarbon-utilizing diazotrophic bacteria from the Arabian gulf coasts, and their potential for oil bioremediation without nitrogen supplementation. Microbial Ecol.

[21] Al-Awadhi H, Al-Mailem D, Dashti N, Khanafer M, Radwan SS (2012). Indigenous hydrocarbon-utilizing bacterioflora in oil-polluted habitats in Kuwait, two decades after the greatest man-made oil spill. Arch Microbiol.

[22] Al-Awadhi H, Dashti N, Kansour M, Sorkhoh NA, Radwan SS (2012). Hydrocarbon-utilizing bacteria associated with biofouling materials from offshore waters of the Arabian gulf. Int Biodeter Biodegr.

[23] Ratledge C, Watkinson I (1978). Degradation of aliphatic hydrocarbons. Developments in biodegradation of hydrocarbons. Vol 1. Essex: applied science.

[24] Rehm H-J, Reiff I (1981). Mechanisms and occurrence of microbial oxidation of long-chain alkanes. Adv Biochem Eng.

[25] Radwan SS, The SNA (1993). Lipids of *n*-alkane-utilizing microorganisms and their application potential. Adv Appl Microbiol.

[26] Al-Sarawi HA, Mahmoud HM, Radwan SS (2008). Pyruvate-utilizing bacteria as potential contributors to the food web in the Arabian gulf. Mar Biol.

[27] Fernandez-Martinez J, Pujalte MJ, García-Martínez J, Mata M, Garay E, Rodríguez-Valeral F (2003). Description of *Alcanivorax venustensis* sp nov and reclassification of *Fundibacter jadensis* DSM 12178T (Bruns and Berthe-Corti 1999) as *Alcanivorax jadensis* comb nov, members of the emended genus *Alcanivorax*. Int J Syst Evol Microbiol.

[28] Engelhardt MA, Daly K, Swannel RPJ, Head IM (2001). Isolation and characterization of a novel hydrocarbon-degrading, gram positive bacterium, isolated from intertidal beach sediments, and description of *Planococcus alkanoclasticus* sp nov. J Appl Microbiol.

[29] Sorkhoh NA, Ghannoum MA, Ibrahim AS, Stretton RJ, Radwan SS (1990). Crude oil and hydrocarbon degrading strains of *Rhodococcus rhodochrous* isolated from soil and marine environments in Kuwait. Environ Pollut.

[30] Santegoeds GM, Ferdelman TG, Muyzer G, Beer DD (1998). Structural and functional dynamics of sulfate-reducing populations in bacterial biofilms. Appl Environ Microbiol.

[31] Altschul SF, Madden TL, Schäffer AA, Zhang J, Zheng Z, Miller W, Lipman DJ, Gapped BLAST (1997). PSI-BLAST: a new generation of protein database search programs. Nucleic Acids Res.

[32] Swofford DL. PAUP*, Phylogenetic analysis using parasimany (* and other methods), version 4b10, Sunderland: Sinauer Association; 1998.

[33] Spectrophotometric LE (1957). Turbidimetric methods for measuring proteins. Methods Enzymol.

