# Supplementary material for: Ability of the So-Called Obligate Hydrocarbonoclastic Bacteria to Utilize Nonhydrocarbon Substrates Thus Enhancing Their Activities Despite their Misleading Name
Source: BMC Microbiol. 2019 Feb 18;19:41. doi: 10.1186/s12866-019-1406-x (PMC6379940; doi:10.1186/s12866-019-1406-x)
Supplement: Supplementary file 1 — Figure S1. Kuwait map showing the water sampling sites (source: d-maps.com: https://d-maps.com/carte.php?num_car=400&lang=en, labeled by Microsoft Office 2007). Figure S2. Comparison of growth qualities for Alcanivorax spp. and Marinobacter spp. from the Arabian Gulf on solid mineral medium containing crude oil or conventional carbon sources (500 mg l− 1) as sole sources of carbon and energy. A, A. borkumensis; B, A. dieselolei; C, A. marinus; D, M. hydrocarbonoclasticus; E, M. vinifirmus . Figure S3. Liquid cultures of individual OHCB from the Arabian Gulf in mineral medium supplied with 500 mg l− 1 peptone. as a sole source of carbon and energy. A, the Alcanivorax spp., 1, A. xenomutans; 2, A. jadensis; 3, abiotic control (not inoculated medium); 4, A. marinus; 5, P. okeanokoites; B, the Marinobacter spp., 1, M. hydrocarbonoclasticus; 2, M. vinifirmus; 3, M. litoralis; 4, abiotic control. The medium turbidity indicates the excellent peptone utilization by the studied OHCB. Figure S4. Standard curve constructed for determination of peptone. Figure S5. GLC profiles showing crude oil consumption by individual OHCB from the Arabian Gulf. Figure S6. Typical GLC-profiles illustrating the enhancement of growth and oil-consumption by representative “OHCB” when treated with nonhydrocarbon substrates. Smaller peaks mean less residual oil in the medium due to higher oil-consumption rates by the tested strain. (PDF 592 kb) [file 12866_2019_1406_MOESM1_ESM.pdf]

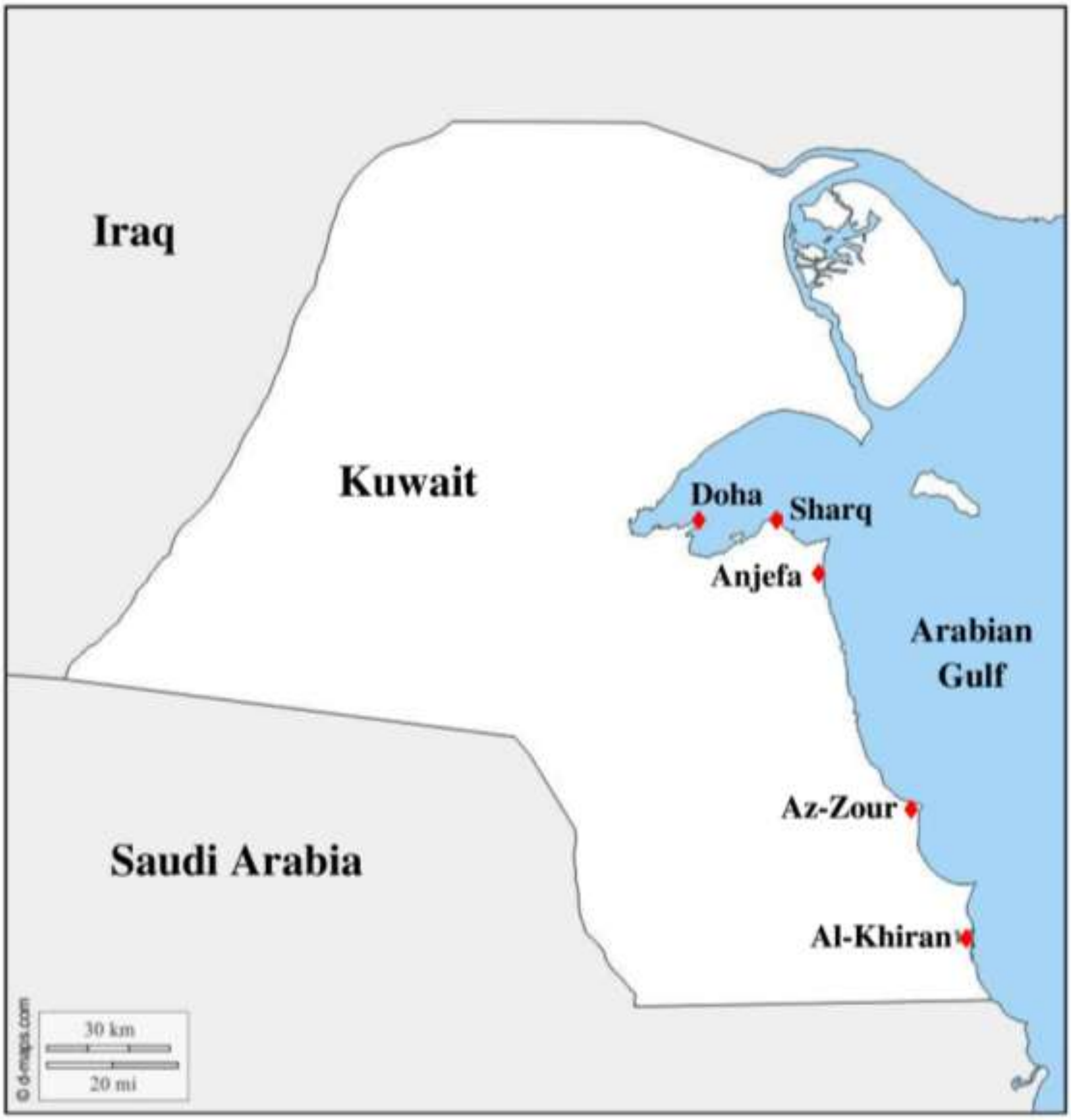

Fig. S1. Kuwait map showing the water sampling sites (source: d-maps.com: https://d-maps.com/carte.php?num_car=400&lang=en, labeled by Microsoft Office 2007).

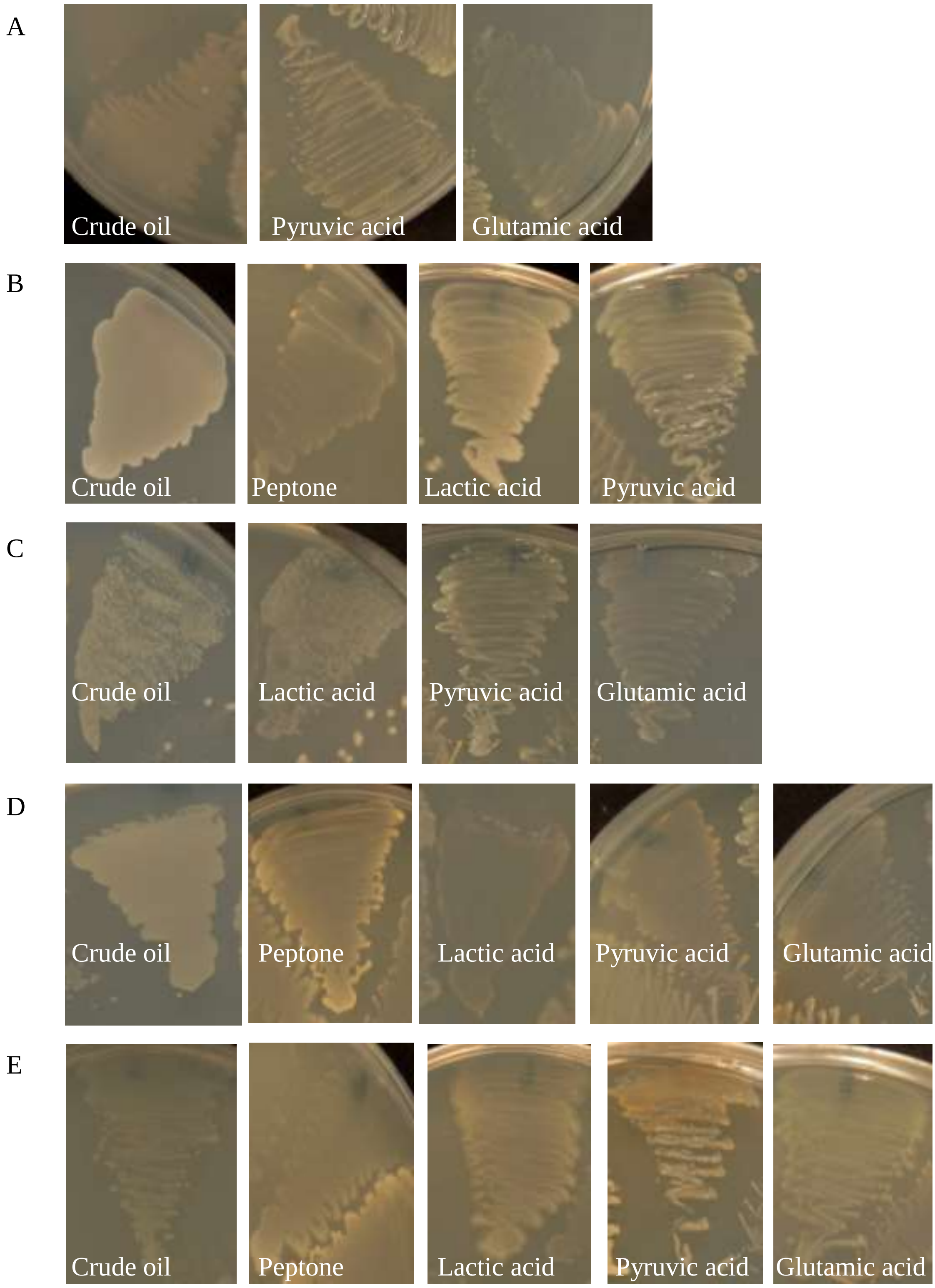

Fig. S2. Comparison of growth qualities for *Alcanivorax* spp and *Marinobacter* spp from the Arabian Gulf on solid mineral medium containing crude oil or conventional carbon sources

(500 mg l^{-1}) as sole sources of carbon and energy. A, *A. borkumensis*; B, *A. xenomutans*; C, *A. marinus*; D, *M. hydrocarbonoclasticus*; E, *M. vinifirmus* .

A

B

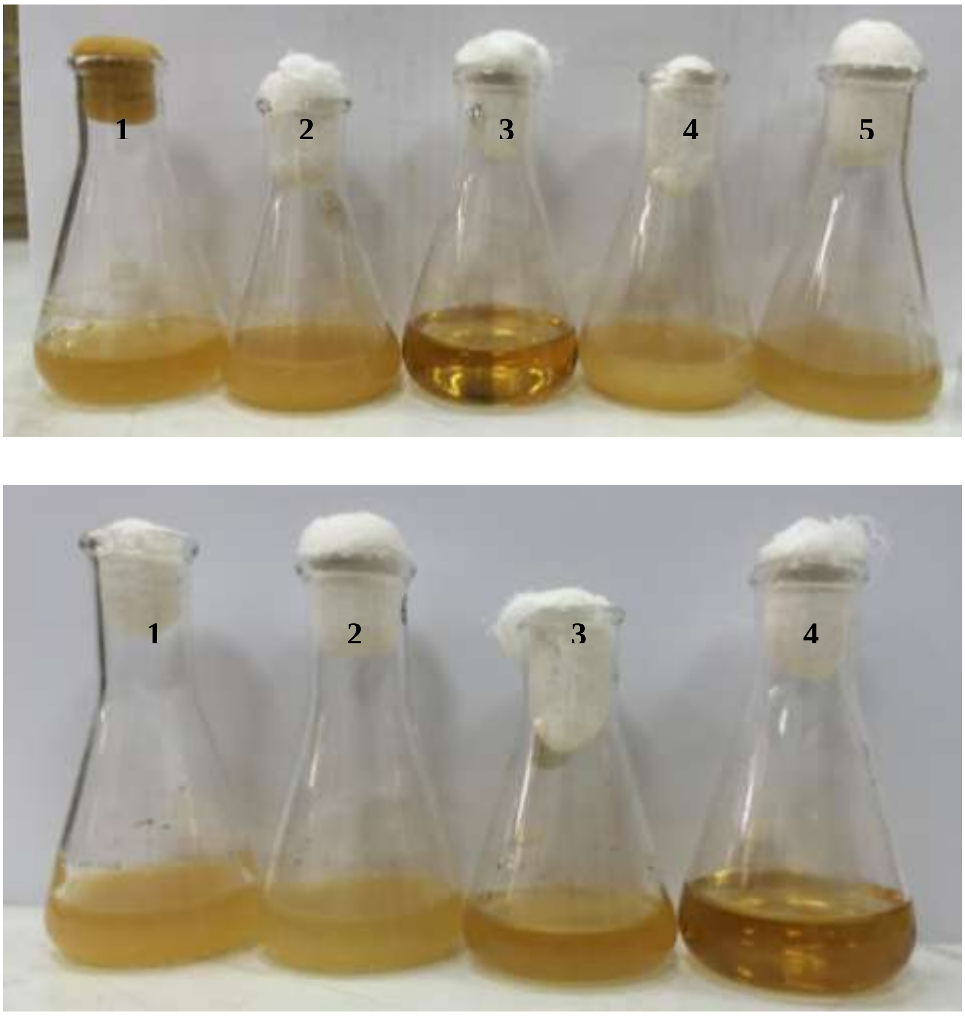

Fig. S3. Liquid cultures of individual OHCB from the Arabian Gulf in mineral medium supplied with 500 mg l^{-1} peptone. as a sole source of carbon and energy. A, the *Alcanivorax* spp, 1, *A. xenomutans*; 2, *A. jadensis*; 3, abiotic control (not inoculated medium); 4, *A. marinus*; 5, *P. okeanokoites*; B, the *Marinobacter* spp, 1, *M. hydrocarbonoclasticus*; 2, *M. vinifirmus* ; 3, *M. litoralis*; 4, abiotic control. The medium turbidity indicates the excellent peptone utilization by the studied OHCB.

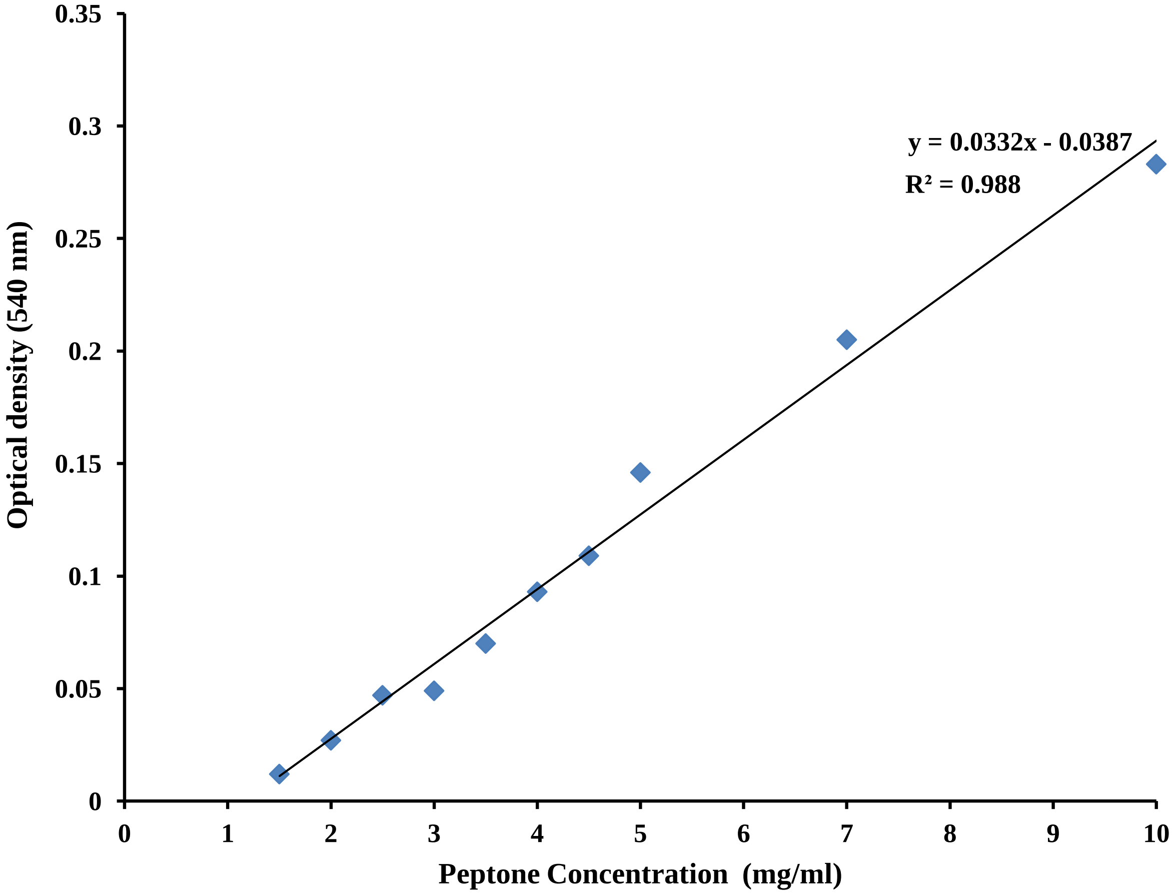

Fig. S4. Standard curve constructed for determination of peptone.

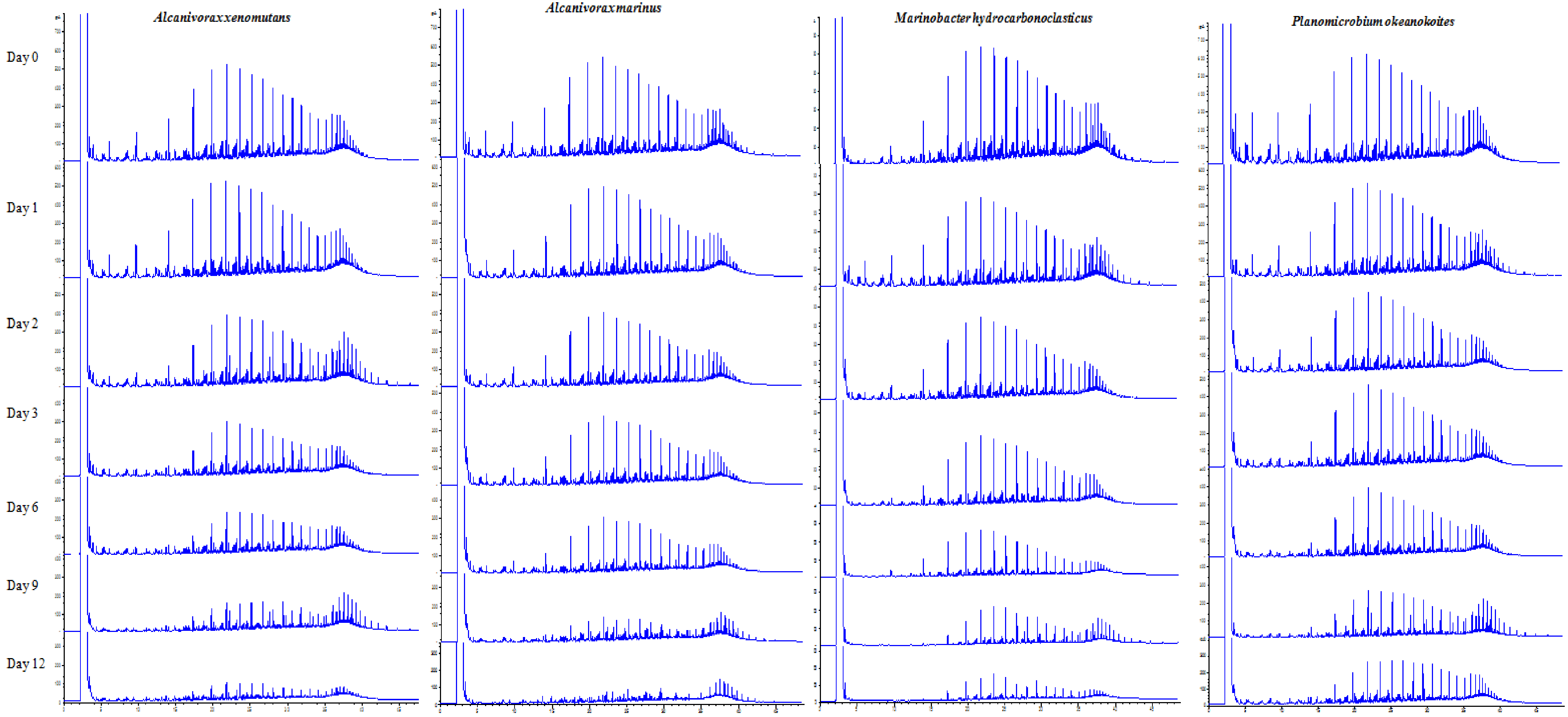

Fig. S5. GLC profiles showing crude oil consumption by individual OHCB from the Arabian Gulf.

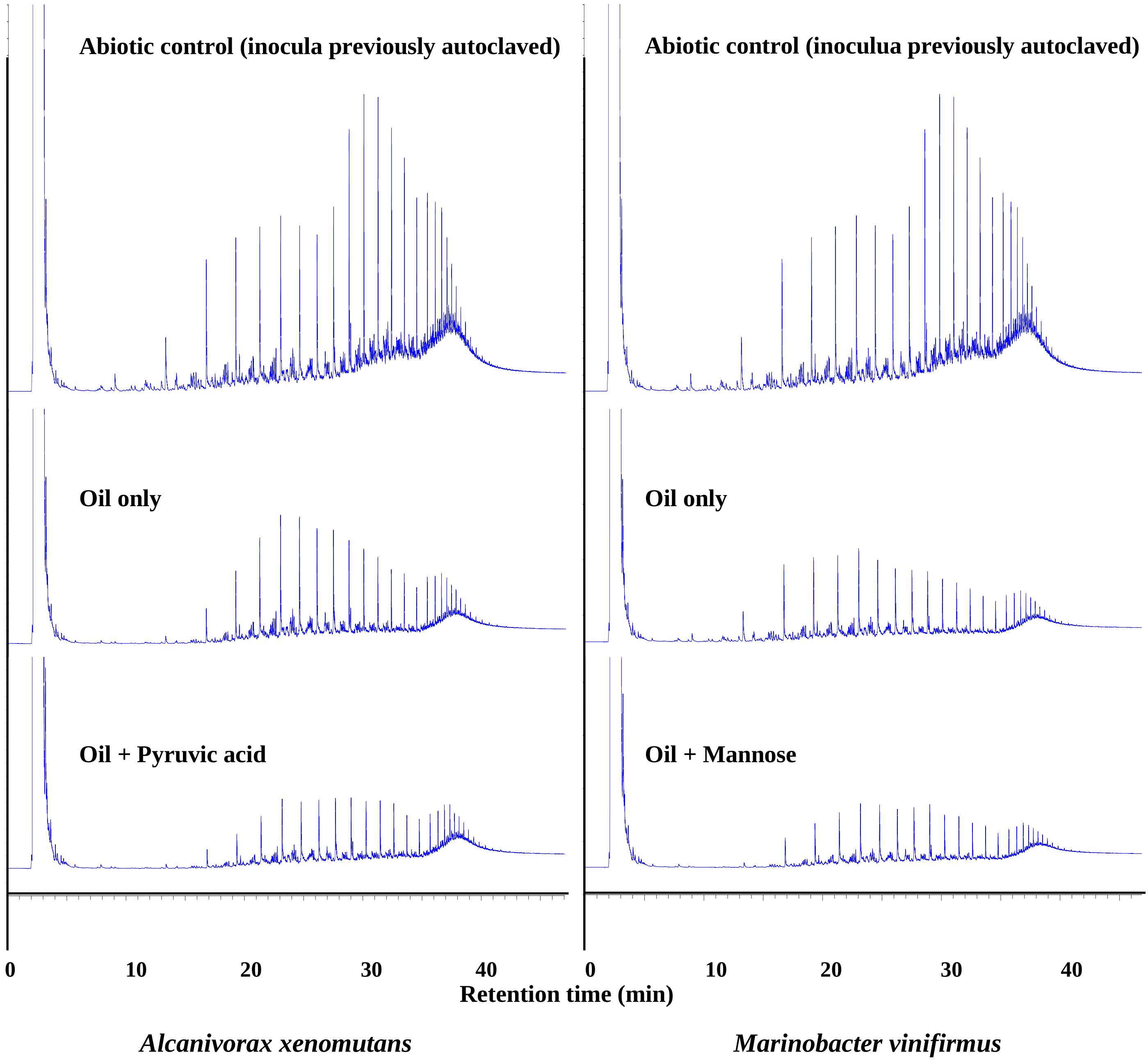
Abiotic control (inocula previously autoclaved)
Oil only
Oil + Pyruvic acid
Abiotic control (inoculua previously autoclaved)
Oil only
Oil + Mannose
0
10
20
30
40
0
10
20
30
40
Retention time (min)
Alcanivorax xenomutans
Marinobacter vinifirmus

Fig. S6.
